# Maize Aflatoxin Accumulation Segregates with Early Maturing Selections from an S2 Breeding Cross Population

**DOI:** 10.3390/toxins5010162

**Published:** 2013-01-15

**Authors:** W. Brien Henry

**Affiliations:** Department of Plant and Soil Sciences, Mississippi State University, 117 Dorman Hall, Box 9555, MS 39762, USA; E-Mail: BHenry@pss.msstate.edu; Tel.: +1-662-325-7483; Fax: +1-662-325-8742

**Keywords:** aflatoxin, GEM Project, maize germplasm, bidirectional selection, *Aspergillus flavus*, maturity

## Abstract

Maize breeders continue to seek new sources of aflatoxin resistance, but most lines identified as resistance sources are late maturing. The vast difference in flowering time makes it hard to cross these lines with proprietary commercial lines that mature much earlier and often subjects the reproductive phase of these resistant lines to the hottest and driest portion of the summer, making silking, pollination and grain fill challenging. Two hundred crosses from the GEM Project were screened for aflatoxin accumulation at Mississippi State in 2008, and a subset of these lines were screened again in 2009. The breeding cross UR13085:S99g99u was identified as a potential source of aflatoxin resistance, and maturity-based selections were made from an S2 breeding population from this same germplasm source: UR13085:S99g99u-B-B. The earliest maturing selections performed poorly for aflatoxin accumulation, but later maturing selections were identified with favorable levels of aflatoxin accumulation. These selections, while designated as “late” within this study, matured earlier than most aflatoxin resistant lines presently available to breeders. Two selections from this study, designated S5_L7 and S5_L8, are potential sources of aflatoxin resistance and will be advanced for line development and additional aflatoxin screening over more site years and environments.

## 1. Introduction

Aflatoxin is a problematic mycotoxin in maize produced throughout the Southern United States and regions of China, Africa and India. Aflatoxin contaminated grain presents problems for growers because, depending upon the year, toxic maize results in significant losses for growers [1,2]. Aflatoxin contaminated grain may result in health problems for both humans and livestock if it enters the food supply, particularly in developing countries [3,4,5]. Few, if any, subsistence farmers have the means or ability to test for aflatoxin, and maize with Aspergillus ear rot and aflatoxin contamination may be consumed by humans. 

Reducing aflatoxin in maize can be accomplished partly by improving cultural production practices of the maize crop [6] and optimizing storage facilities of the harvested maize grain [7], but one of the most widely accepted means of lowering grain aflatoxin is breeding for improved maize lines with reduced grain aflatoxin accumulation [8]. Breeding efforts to lower grain aflatoxin accumulation have resulted in the release of six Mississippi lines Mp313E, Mp420, Mp715, Mp717, Mp718 and Mp719 [9,10,11]. Mp718 and Mp719 were released recently because of improved agronomic traits [12]. These two lines were derived from crosses between a resistant line, Mp715 and Va35. Resistant lines 13 days earlier and at least 15% shorter than Mp715 were identified through multiple generations of self pollination and aflatoxin screening. Earlier flowering times and improved agronomic characteristics, like shorter plant and ear heights, will increase the likelihood of these resistant lines being used in crosses with elite breeding material from proprietary commercial maize breeding programs [12]. 

An additional strategy for lowering grain aflatoxin is biological control [13,14]. These biocontrol agents used together with better agronomic practices and hybrids with better characteristics, including genetic resistance to aflatoxin accumulation, should improve the likelihood of reduced aflatoxin in the grain. 

Additional lines with moderate aflatoxin resistance have been released by researchers in Georgia and Texas. USDA-ARS researchers at Tifton, GA released GT603 with improved combining ability compared with previously released Georgia lines and maturity 10 days earlier than Mp313E [15]. GT603 was developed out of population GT-MAS:gk (PI 561859) with traits that contribute to lowered aflatoxin accumulation, like pericarp wax, antifungal proteins and early flowering [15]. Texas A&M researchers released three lines, Tx736, Tx739 and Tx740, with both favorable agronomic and reduced toxin characteristics compared with other commercial lines [16]. Tx736 was derived from the aflatoxin resistance source Tx772 crossed to T246. A first generation backcross to Tx772 followed by multiple generations of selfing and aflatoxin screening resulted in desirable ear characteristics, like enhanced kernel quality, grain fill, flowering synchrony and low aflatoxin accumulation [16]. Tx739 and Tx740 were selected from an S3 population for low aflatoxin accumulation, but also short plants and ear placement, desirable kernel texture and flinty yellow/orange color and adaptation to a Texas environment with respect to flowering synchrony and maturity [16]. Additional sources of resistance are currently being sought, and both conventional and marker-assisted breeding efforts are being used to develop germplasm lines at the USDA-ARS location at Mississippi State [17]. 

Potential new sources of mycotoxin resistance are available from the Germplasm Enhancement of Maize (GEM) Project. The GEM Project is a collaborative effort of USDA-ARS, universities, private industry, international agricultural research centers and non-governmental organizations (NGO) to broaden the germplasm base of maize [18,19]. The GEM Project’s goal is the development of adapted germplasm for the US Corn Belt utilizing tropical and temperate exotic germplasm. Li *et al.* [20] reported that substantial variability existed among maize breeding crosses for aflatoxin resistance from germplasm evaluated from the GEM Project. It was suggested that the most resistant breeding crosses be used to develop new inbred lines. 

Germplasm from the GEM Project was evaluated with the following two objectives: (1) to identify new sources of germplasm with resistance to aflatoxin accumulation; and (2) to identify sources of resistance with favorable agronomic characteristics, specifically early maturity. 

## 2. Results and Discussion

**Figure 1:**
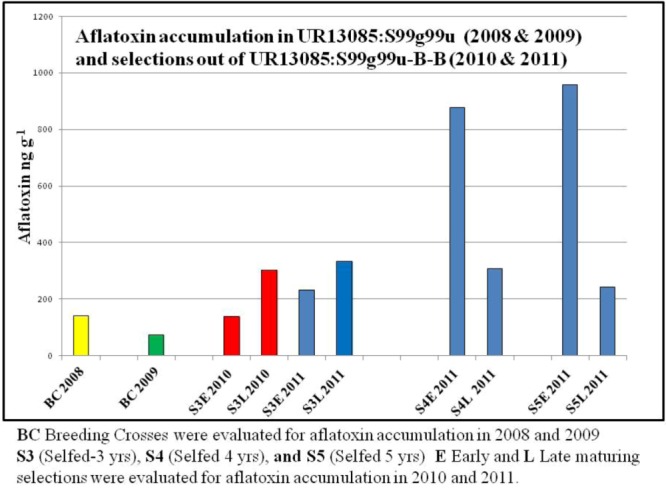
Aflatoxin accumulation in early (E) and late (L) maturing selections in S3 through S5 stages of advancement out of the breeding cross UR13085:S99g99u.

UR13085:S99g99u was selected for further investigation from among 200 breeding crosses, including known resistant and susceptible checks that were screened in 2008, because it exhibited low levels of grain aflatoxin accumulation (Figure 1). 

Repeated aflatoxin evaluation alongside known resistant and susceptible checks in 2009 and the resulting low aflatoxin levels confirmed that this breeding cross may hold potential as a source of resistance to aflatoxin accumulation (Figure 1). With respect to Objective #1 of our study, this breeding cross does appear to be a potential source of aflatoxin resistance. 

**Figure 2:**
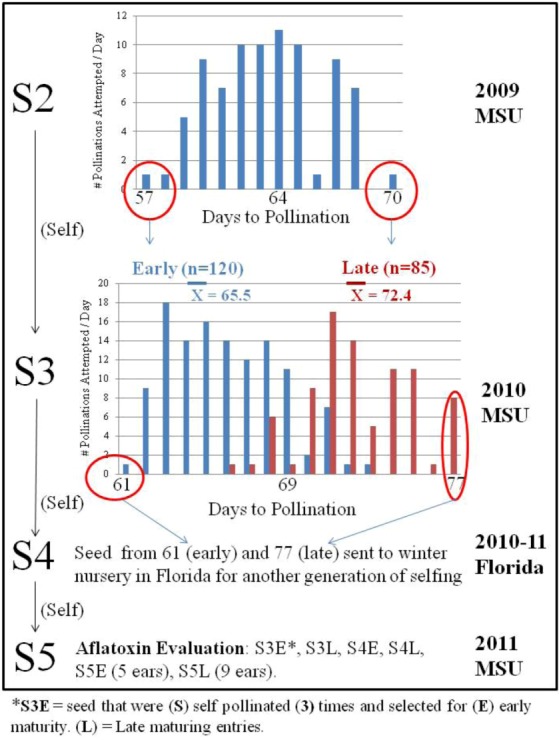
Line development out of a UR13085:S99g99u-B-B breeding cross population with S3 and S4 selections based upon maturity.

An S2 breeding population from this same germplasm source, UR13085:S99g99u-B-B, was evaluated in 2009. The UR13085:S99g99u-B-B breeding population exhibited a wide range of maturity in our 2009 nursery, ranging from the earliest at 57 days to pollination (DTP) and the latest at 70 DTP (Figure 2). 

Because most aflatoxin resistance sources are late maturing, it would be beneficial to identify resistance in earlier maturing germplasm to facilitate breeding with elite commercial lines [12]. Selections from the extreme tails of the maturity distribution were chosen in 2009 and 2010 at MSU with the goal of capturing aflatoxin resistance in early germplasm. It was not possible to base selections upon maturity data of the S4 material in the 2010-11 Florida nursery; these ears were advanced to S5 with another generation of self pollinations. One generation of bidirectional selection in 2009 moved the mean maturity date of the early and late populations grown in 2010 a week apart (Figure 2). The tails of the distribution (earliest of the early selections and latest of the late selections) were 16 days apart in 2010, compared with only 13 days apart in 2009 (Figure 2). In addition to changes in DTP, other agronomic characteristics were affected by selecting for maturity.

Individual S5 ears from the early and late selections were used as entries in the 2011 aflatoxin evaluation (Table 1). 

**Table 1:** Agronomic characteristics and aflatoxin accumulation for early and late selections out of an S2 breeding population UR13085:S99g99u-B-B in 2011.

Treatment §	Plant Height ¥		Ear Height		Mid Tassel €		Mid Silk		Ear Rot ₫		Field Rating		Aflatoxin †	
S5_E1	129	Fg ‡	47	e	55.0	fg	58.7	fg	4.0	a–e	5.0	b–e	2012	a
S5_E2	141	ef	47	e	55.3	fg	56.7	g	6.0	ef	4.0	de	1011	a–c
S5_E3	123	g	43	e	54.7	f–h	57.0	fg	3.0	a–c	6.3	bc	468	a–e
S5_E4	122	g	45	e	54.7	f–h	57.3	fg	5.0	c–e	3.7	e	890	a–d
S5_E5	135	fg	47	e	56.7	ef	58.3	fg	3.7	a–e	4.3	c–e	406	b–e
S5_L1	122	g	61	d	59.7	cd	62.0	de	7.3	f	3.3	e	1361	ab
S5_L2	164	cd	67	b–d	61.5	bc	66.3	b	5.3	c–e	6.3	bc	183	d–e
S5_L3	177	a–c	68	b–d	63.3	b	67.0	b	1.7	a	4.7	b–e	412	b–e
S5_L4	167	b–d	70	a–d	61.7	bc	67.0	b	2.3	ab	3.3	e	306	b–e
S5_L5	180	ab	71	a–c	61.7	bc	65.7	b	4.0	a–e	4.0	de	306	b–e
S5_L6	-	-	-	-	-	-	-	-	-	-	9.0	a	-	-
S5_L7	167	b–d	68	b–d	62.0	bc	65.3	bc	2.0	a	4.7	b–e	115	e
S5_L8	184	a	73	ab	62.3	b	67.7	b	3.3	a–d	4.0	de	132	e
S5_L9	-	-	-	-	-	-	-	-	-	-	9.0	a	-	-
S4_E	122	g	39	e	52.3	gh	56.0	g	4.7	b–e	6.0	b–d	878	a–d
S4_L	164	cd	68	b–d	62.0	bc	65.3	bc	4.7	b–e	4.7	b–e	308	b–e
S3_E	169	b–d	69	a–d	53.3	gh	56.7	g	4.0	a–e	3.3	e	232	c–e
S3_L	155	de	63	cd	58.7	de	62.7	cd	4.7	b–e	5.0	b–e	332	b–e
Mp317	167	b–d	78	a	66.3	a	73.3	a	-	-	6.7	b	-	-
LSD	15		10		2.6		2.8		2.3		2.3		-	-

§ Treatments include selections in various stages of advancement, ranging from three to five generations of selfing. Selections based upon maturity are designated by early (E) or late (L). Multiple S5 early ears were used as entries, designated S5_E1 through S5_E5. Multiple S5 late ears were designated similarly, S5_L1 through S5_L9; ¥ Plant Height and Ear Height were recorded in centimeters (cm); € Tasseling and Silking data were recorded in days from planting. Mid tassel/silk is the date at which half the plants in a row exhibited exposed tassels/silks. Full tassel/silk is the date at which every plant in the row exhibited tassels/silks; ₫ Ear Rot ratings were (1–9), with 9 representing a fully rotten ear and 1 representing a clean ear with no rot. Field Ratings were scored similarly (1–9) with respect to % emergence, vigor, greenness and stalk strength (1 was best, and 9 was worst); ‡ Means in a column followed by the same letter do not differ at *p* < 0.05 (Fisher’s Protected LSD); **†** Means for aflatoxin concentration (ng g^−1^) were transformed [ln (*y* + 1)] before statistical analysis, and tests for significance performed on transformed means prior to conversion back to the original scale. No ears were present on S5_L6, S5_L9 and Mp317; therefore, no aflatoxin data are shown.

Variability remains within the early (S5_E1 through S5_E5) and late (S5_L1 through S5_L9) selections in the most advanced S5 material; however, on average, the late material was about 25% taller with higher ear placement than the early material (Table 1). Additionally, tasseling and silking occurred approximately one week sooner in the early material than in the late material (Table 1). Ear rot and in-field ratings were not influenced by maturity. 

Concerning objective # 2, two years of selecting a limited number of ears for maturity did influence the mean DTP of the early and late populations (Figure 2). Maturity, at least in the limited, early material that we selected, appears to be associated with grain aflatoxin accumulation. The mean toxin accumulation of the early entries was almost 4x that of the late entries. This is not entirely unexpected because mycotoxin resistance in the GEM material is assumed to come from the exotic component of the breeding material, which is a late temperate flint population from Uruguay. The GEM breeding crosses resulting from exotic accessions crossed to US Corn Belt inbreds were made to provide maize breeders germplasm for development in non-tropical environments. Selected progenies can therefore be evaluated for various traits, such as mycotoxin accumulation and ear rot. Many late, aflatoxin resistant lines, like Mp715 and Mp317, often mature so late in the summer that excessive heat and/or drought limit seed production. This was the case in 2011 for Mp317, which was included in the trial as a resistant check (Table 1). Williams and Windham [12] provide midsilk data (number of days from planting until silks emerge on 50% of the plants in a row) from replicated field trials averaged over eight environments of 85, 81 and 77 for the aflatoxin resistant inbreds Mp715, Mp313E and Mp717, respectively. Two other inbred lines with resistance to aflatoxin accumulation, Mp494 and Mp317, have demonstrated aflatoxin resistance in prior studies [21], and in un-replicated nursery-row observations, they are similarly late maturing. 

Although the early selections from this study performed poorly for aflatoxin accumulation, two of the late, S5 selections, S5_L7 and S5_L8, generated low aflatoxin accumulation data of 115 and 132 ng g^−1^, respectively. Regarding Objective # 2 of this study, although some selections derived from UR13085:S99g99u-B-B were designated as “late,” these two selections, S5_L7, 65 Days to Midsilk (DTM) and S5_L8, 68 DTM, were considerably earlier than traditional aflatoxin resistance sources, with maturity ranging from approximately 75 to 85 DTM [12]. For comparison, toxin data (averaged over six growing seasons and presented in ng g^−1^) for the two most recently released lines were as follows: Mp718 = 223 and Mp719 = 74. Toxin data for the commonly used resistance sources were similar: Mp717 = 125, Mp715 = 112 and Mp313E = 65 [12]. Aflatoxin levels in 2011 for entries S5_L7 and S5_L8 were 115 and 132 ng g^−1^, respectively, although for only one year and one environment with the S5’s, it compares very favorably to historically resistant aflatoxin lines. 

If seeking aflatoxin resistance in the earliest material were an absolute priority, sib-mating strategies could be instituted in an attempt to break linkage barriers between aflatoxin resistance and maturity. We could also increase the numbers of ears evaluated and the number of selections made from the early end of the maturity distribution. Screening lines earlier in the selection process for aflatoxin accumulation advancing only the earliest and most resistant lines might also result in earlier resistance sources. It should also be noted that because of a wet spring, the 2011 planting date was later than usual, providing seedlings with more heat units early and potentially reducing the number of days to maturity. Although Mp317 did not produce grain, for comparison, midsilk at 73 days was approximately seven days earlier than what we would expect in a “normal” growing season, suggesting that our maturity data from the other entries might also be somewhat earlier than what may be seen in another environment or site-year. 

## 3. Experimental Section

### 3.1. Planting Dates and Experiments

Multiple experiments to evaluate breeding crosses and selected material out of these crosses for aflatoxin accumulation were conducted at the Rodney Foil Research Facility at Mississippi State University (MSU) from 2008 through 2011. The 2008 and 2009 aflatoxin evaluations of breeding crosses were planted on 15 and 29 April, respectively. The 2010 aflatoxin evaluation of early (E) and late (L) selections from UR13085:S99g99u-B-B S2 families was planted April 14, 2010. The 2011 study included early and late selections from UR13085:S99g99u-B-B at the S3 (three generations of selfing), S4 (four generations of selfing) and S5 (five generations of selfing) stages of advancement and was planted on May 10, 2011. 

### 3.2. Selection for Maturity

Concurrent to the 2009 field trials for aflatoxin accumulation, a block of approximately 100 plants (20 plants per row, 5 rows) of the breeding population UR13085:S99g99u-B-B was grown in our 2009 nursery. These plants were self-pollinated, and the number of days from planting until pollination, DTP (Days to Pollination), was recorded for each pollination. Attempted pollinations within this block ranged from 57 to 70 DTP. Seed from the two earliest (57 and 58 DTP) and the latest (70 DTP) ears were selected and used as seed sources to plant similar “early” and “late” blocks of 160 plants per block the following year. In 2010, the earliest ear of the S3 generation from the early population (61 DTP) and the latest ear from the S3 generation late population (77 DTP) were selected for advancement in the 2010–2011 winter breeding nursery. Seed from the early and late ears were planted to single rows, which were selfed in Florida in 2010/2011. S5 (selfed for five generations) ears received from the Florida nursery from the early and late rows were used as seed sources for entries in a replicated field trial in 2011. There were five early ears and nine late ears in the 2011 aflatoxin trial. In addition to the S5 early and late material, remnant seed from the S4 early and late and remnant seed of the S3 early and late material were included as additional entries for comparison. 

### 3.3. Germplasm

Seed of the initial breeding crosses was received from the GEM Project in 2008. Two hundred crosses were evaluated for aflatoxin accumulation. One of the top performing breeding crosses was UR13085:S99g99u, a cross involving an exotic Uruguay accession, UR13085 (PI 583927), and two US stiff stalk lines, S99g, and 99u. The Uruguay accession is an orange flint population from the race Cateto Sulino. The exotic source represents 25% of the pedigree, and S99g and S99u represent 25% and 50% of the pedigrees, respectively. S99g is a public inbred line N28, known for its extensive root system. Superior performing hybrids constructed from this line were identified in older, unglaciated soils in which the extensive roots of N28 were able to penetrate the hardpan [22]. The other stiff stalk component of this breeding cross is S99u, which is CUBA117:S1520-156-001-B-B-B. A breeding cross with a closely related pedigree, CUBA117:S15-101-001-B-B-B-B-B/GEMN-0140, was recently identified as a potential source of aflatoxin resistance [23], suggesting that the CUBA117 portion of this pedigree may contribute to reduced aflatoxin accumulation. 

Self-pollination and selection of this breeding cross to the S2 generation were designated as UR13085:S99g99u-B-B, where each B represents one generation of selfing. This breeding cross is one among many potential sources of resistance evaluated at the USDA-ARS location at Mississippi State. The seed lot from which maturity-based selections were made was an S2 breeding population, UR13085:S99g99u-B-B, which was also received from the GEM project in 2009. All GEM material was derived from self-pollination in accordance with GEM protocol using the pedigree breeding method. Phenotypic traits and protocols for breeding and writing pedigrees can be found on the GEM Project website [24].

### 3.4. Plot Size and Management

Entries in the 2008–2011 aflatoxin evaluations were planted in 5.1 m long single-row plots, with rows spaced 0.97 m apart and thinned to 20 plants per row in a randomized complete block with three replicates. Fertilizer and herbicides were applied in accordance with standard local production practices, and at no time throughout the season was weed pressure or fertility limiting for crop growth. All years experienced periodic drought conditions, and supplemental furrow irrigation was applied to the aflatoxin evaluations once in 2008 and 2010 and multiple times in 2009 and 2011 to assure that there would be enough grain to harvest. Rows in the breeding nursery were managed similarly to the aflatoxin trials with respect to row spacing, plant population, fertility and weed control. The breeding nursery was irrigated extensively in 2009 and 2010. 

### 3.5. Fungal Inoculum and AflatoxinPreparation

*A. flavus* isolate NRRL 3357 has produced high levels of aflatoxin in maize grain in prior studies [25,26,27] and was used to prepare the inoculum in these experiments. Inoculum was increased according by growing *A. flavus* on sterile maize cob grits (size 2040, Grit-O-Cobs^®^, The Andersons, Maumee, OH, USA) in 500-mL flasks, as described by Windham *et al.* [28]. Each flask contained 50 g of grits and 100 mL of sterile, distilled water and was incubated at 28 °C for 3 weeks. Conidia in each flask was washed from the grits using 500 mL sterile distilled water containing 0.1% Tween 20 per liter and filtered through four layers of sterile cheesecloth. A tree marking gun was used to deliver a 3.4-mL spore suspension containing 3 × 10^6^ *A. flavus* conidia at 7 days after midsilk. The solution was injected underneath the husks near the middle of the ear. 

Ears were harvested approximately 60 day following inoculation and placed in a forced air drier at 38 °C for 5 to 7 days. Following drying, the ears from each plot were bulked together, shelled, mixed thoroughly and ground with a Romer Mill (Union, MO, USA). Aflatoxin concentration was determined with the Vicam Aflatest (Watertown, MA, USA), which detects aflatoxin levels as low as 1 ng g^−1^. 

### 3.6. Statistical Analysis

All statistical analyses were conducted using the SAS software package (version 8.2; SAS Institute Inc., Cary, NC, USA). Logarithmic transformation [log (*y* + 1)] was used on all aflatoxin data to stabilize the variance. Data were analyzed with the PROC GLM procedure, and means were separated using Fisher’s protected least significant difference test at *p* = 0.05. Aflatoxin data are reported as geometric means (antilog of the logarithmic mean). 

## 4. Conclusions

The breeding cross, UR13085:S99g99u, was identified as a potential source of aflatoxin resistance through two years of screening in replicated field trials. The majority of aflatoxin resistant lines presently available to maize breeders are late maturing, and it would be beneficial to identify earlier maturing sources of germplasm with aflatoxin resistance. Selections out of an S2 breeding population from this same germplasm source, UR13085:S99g99u-B-B, were advanced with respect to maturity. Early maturing selections performed poorly for aflatoxin accumulation; however, two S5 lines exhibited low grain aflatoxin scores and favorable maturity data, even though they were designated as “late” in this study. These lines are promising sources of aflatoxin resistance for both mycotoxin accumulation and early maturity and will be advanced for line development and additional testing in subsequent years. 
